# Paediatric nurses’ general self-efficacy, perceived organizational support and perceived professional benefits from Class A tertiary hospitals in Jilin province of China: the mediating effect of nursing practice environment

**DOI:** 10.1186/s12913-019-4878-3

**Published:** 2020-01-03

**Authors:** Linan Cheng, Yajuan Cui, Qian Chen, Yansheng Ye, Yingchun Liu, Fengying Zhang, Weiyan Zeng, Xiuying Hu

**Affiliations:** 1 West China Hospital/West China School of Nursing, Sichuan University, No. 37, Guoxue Alley, Chengdu, Sichuan 610041 China; 2 Department of Hepatobiliary Pancreatic Surgery, First Hospital Bethune, Jilin University, No. 71, Xinmin Street, Changchun, Jilin 130021 China; 3 Quality Management Department, Pengzhou People’s Hospital, No. 255, South Third Ring Road, Pengzhou, Sichuan 611930 China

**Keywords:** General self-efficacy, Perceived organizational support, Perceived professional benefits, Nursing practice environment, Mediating role

## Abstract

**Background:**

General self-efficacy is considered one of the most influential parameters affecting the quality of clinical practice and nurses’ perceived professional benefits (NPPB). Perceived organizational support (POS) is regarded as being central in understanding job-related attitudes, and it is important to enhance POS for nurses to maintain their current employment. NPPB can further reduce nurses’ job burnout and turn-over intention. Many studies have explored the relationships among general self-efficacy, POS, nursing practice environment (NPE) and NPPB. However, a moderating effect of NPE has not been fully explored in nurses, especially among paediatric nurses.

**Methods:**

A descriptive cross-sectional study was conducted from July to October 2018 with 300 paediatric nurses from 3 Class A tertiary hospitals in Jilin Province. The respondents completed the General Self-Efficacy Scale, Perceived Organizational Support Scale, Practice Environment Scale and Nurses’ Perceived Professional Benefits Scale. The data were analyzed using path analysis and SPSS (version 23.0, IBM).

**Results:**

General self-efficacy and POS were significantly positively associated with NPPB, which showed that the model had a good fit to the data. NPE was found to play a partial mediating role between POS and NPPB and also had a complete mediating role between general self-efficacy and NPPB.

**Conclusions:**

The results suggest that general self-efficacy indirectly influences NPPB, and POS directly and indirectly influences NPPB by NPE. Effective measures should be taken to improve nurses’ practice environment in hospitals to raise nurses’ enthusiasm and confidence in their work.

## Background

A contradiction between the supply and demand of children's medical services has become more prominent due to the “two-child” policy in China [1]. Meanwhile, paediatric nurses may be experiencing stress at a much higher level than nurses in other specialities, especially in various kinds of stressful and fast-paced environments while caring for patients and their family members with complex healthcare needs [2]. As we all know, paediatrics is known as the “dumb department”.

Paediatric nurses are required to face complex work environments, such as inappropriate children, noisy work environments, excessive workloads, demanding operational skills and high-risk work violence [3].

All of these abovementioned factors could increase nurses’ fatigue, burnout, callousness, indifference, and even turnover [4]. In contrast, the imbalance in the supply of paediatric nurses has become even more prominent. According to a previous report, approximately 70.7% of paediatric nurses in China have suffered from workplace violence, which has seriously affected their self-esteem and professional identity, resulting in job burnout, which has brought adverse outcomes to the entire nursing industry. Researchers have done a substantial amount of work and have found that NPPB are an effective mechanism to stimulate the inner potential of nurses [5]. NPPB refer to a positive emotional experience from the nursing practice. Moreover, nurses can perceive the gains and benefits brought by their profession in the process of the nursing practice and agree that the nursing profession could promote their overall growth based on positive psychology’s theory [6]. Studies have identified a correlation between well-being and perceived professional benefits [7, 8]. When nurses perceive a sense of achievement from the occupation they are engaged in, they are likely to have a sense of job satisfaction, which in turn could address the current shortage of nurses and ensure an adequate workforce of nurses [9, 10].

Self-efficacy is an evaluation of the capability to perform a certain task and the expectation of being able to successfully perform certain behaviours, and it can change as a result of learning, experience, and feedback [11, 12]. Several studies have evaluated the effect of self-efficacy in maintaining an optimistic attitude, reducing job burnout and increasing positive emotion [13, 14]. Therefore, we assume that there may be a certain relationship between self-efficacy and NPPB. In this study, NPE refers to an environment that enables nurses to gain more autonomy, responsibility and work control through authorization when providing nursing services [15]. Some researchers have reported that self-efficacy can improve an individual's confidence to provide nursing practice in a complex situation [16, 17].

Researchers have demonstrated that the nursing practice environment also significantly affects general self-efficacy (*P*<0.01) [13]. Nursing practice environment and general self-efficacy have positive effects on nurses’ intrinsic satisfaction (*P*<0.01) [14]. Other researchers have also reported that nursing practice environment and turnover intention are negatively correlated (*P* < 0.01) [18]. Another study has shown that a perfect nursing practice environment can improve nurses’ job satisfaction and nursing service quality [19]. Numerous studies have also tested the relationships among self-efficacy, NPE and relevant benefits; however, self-efficacy needs to produce corresponding effects under certain environments or conditions, and a mechanism of NPE has not yet been fully explored. Therefore, the first purpose of this study is to explore the effects of self-efficacy, NPE and relevant benefits.

Perceived organizational support (POS) refers to employees “global beliefs concerning the extent to which the organization values their contributions and cares about their well-being” [20], which is regarded as a psychological agreement that the employee makes with the enterprise [21]. Studies have shown that POS will emerge when performance-reward expectations are mutual between managers and employees [22]. When POS and the needs of compliments and recognition are met, nurses will have positive emotional attitudes towards nursing and therefore make efforts to achieve organizational goals [23]. A growing body of research demonstrates that POS can increase organizational commitment and work performance and reduce turnover and burnout [24, 25]. Wand et al. [26] reported that POS had a positive relationship with perceived professional benefits, which means that nurses with a higher POS had higher NPPB (*P*<0.01). Yuan [27] obtained the same conclusion (*P*<0.05). Currently there are more theoretical studies than intervention literature. Our previous research found a relationship between NPE and POS (*P*<0.01) [28, 29]. It could be helpful to explore how POS intervenes to produce practical effects in nursing practice. Therefore, the second purpose of this study is to find the relationships among POS, NPE and NPPB.

Previous literature have mostly employed multiple logistic or linear analyses to explore the factors associated with NPPB. However, a study on the association between GSE, POS, NPE and a multidimensional variable such as NPPB would have possible limitations on the results in terms of the interpretation and application. Therefore, given that path analysis is an established type of structural equation modelling analysis that simultaneously estimates and tests the directional relationship of a model and is an effective way to identify the direct, indirect, and total effects among variables with other multivariate statistical models [30, 31], the purpose of this study was to develop and test a hypothesized model that uses path analysis to explore the relationships among GSE, POS, NPPB and NPE; the direction and the size of the relationship between the variables; and if the variables have indirect or direct effects.

## Methods

### Design

A cross-sectional design was used in this study.

### Model development

Based on previous literature and the causal pathway model, we developed a theoretical model that explains mechanisms underlying the associations among GSE, POS, NPE and NPPB. Our theoretical model predicts that self-efficacy and POS have positive effects in terms of maintaining an optimistic attitude, reducing job burnout and nurses’ intrinsic satisfaction [14, 23]. Self-efficacy and POS can directly reinforce NPPB that can be indicated by positive emotional needs, and also can indirectly improve NPPB by a perfect nursing practice environment that can be indicated by perfect managerial support for nursing care, good doctor-nurse relations, nurses engaging in decision making, adequate staffing and resource and the basis of high-quality nursing services. As a result of a perfect nursing practice environment, individuals may have better NPPB. Our theoretical model also predicts that better NPPB also can lead to higher higher levels of GSE and POS.

### Sample

A convenience sample of paediatric nurses (including all the paediatric points of care whether emergency, critical care, or inpatient wards) was recruited from 3 Class A tertiary general public hospitals in Jilin Province from July 2018 to November 2018. The hospitals in the sample are university-affiliated hospitals with more than 500 beds integrating medical service, education, research, prevention, health care, and recovery. The sample size for each hospital is 102,98 and 100, and the baseline of these hospitals is essentially the same in terms of working conditions, management, economic income, hospital development, and professional structure. Ethical approval was obtained from an independent research ethics committee in China (ethics number: 2018-554). Participants that met the following inclusion criteria were included in this study: a) those who knew about and agreed to participate in the study; b) on-the-job clinical paediatric nurses with an RN license of the People's Republic of China; and c) those who had worked more than 1 year. The following exclusion criteria were used in this study: a) nurses who were currently completing their internship and/or in training at other hospitals; b) nurses who, during the investigation, had further studies to complete or needed to take sick leave; and c) nurses who were not on duty during the investigation. We carried out a strict level of quality control. Three trained research assistants were sent to collect data in the hospitals. After explaining the purpose and significance of the study, all participants were told that their responses were completely anonymous, and participants declared that their responses were not affected by any power or person according to the informed consent. Respondents had 15~25 minutes to complete the questionnaires. Overall, 320 questionnaires were distributed. After eliminating invalid questionnaires, 300 questionnaires were suitable for statistical analysis (response rate of 92.3%). It has been previously determined that more than 200 samples should be obtained for path analysis [32]. Therefore, the inclusion of 300 paediatric nurses was consistent with this rule.

### Assessment tools

#### General self-efficacy scale

The General Self-Efficacy Scale was developed by German psychologist Schwarzer in 1981 [33]. Based on the original version, the scale was translated and revised by Wang CK for the Chinese version of the General Self-Efficacy Scale [34]. The scale includes 10 items. On a 4-point scale, the responses ranged from 1 (incorrect) to 4 (completely correct), and the total score was between 10 and 40 points. A higher score implies stronger general self-efficacy. The questionnaire is a reliable tool to measure nurses’ general self-efficacy, and the Cronbach’s alpha coefficient was 0.871.

#### Perceived organizational support scale

The Perceived Organizational Support (POS) Scale was developed by Eisenberger [20] and compiled by Chen ZX [35, 36]. Zuo HM [24] appropriately modified the original scale for nurses. The scale contains 13 items, including 2 dimensions, namely, emotional support (10 items) and instrumental support (3 items). On a 5-point scale, the responses ranged from 1 (quite inconsistent) to 5 (quite consistent), and the total score was between 13 and 65 points. A higher score implies a stronger POS. The Cronbach’s alpha coefficient was 0.921.

#### Nurses’ practice environment scale

The Nursing Practice Environment Scale (NPE) was developed by Lake [37] according to the Nursing Work Index Revised (NWI-R). The Chinese version of the NPE was translated and validated by Wang L. [38] The scale contains 31 items, which are divided into 5 dimensions, including nurse participation in hospital affairs (9 items), staffing and resource adequacy (4 items), the basis of high-quality nursing services (10 items), nurse manager ability and leadership style (5 items) and doctor-nurse cooperation (3 items). On a 4-point scale, the responses ranged from 1 (complete nonconformity) to 4 (complete conformity), and the total score was between 31 and 124 points. A higher score implies a better NPE. The Cronbach’s alpha coefficient was 0.91. The sub-scale coefficients ranged from 0.67 to 0.79.

#### Nurses’ perceived professional benefits scale

The Nurse’s Perceived Professional Benefits Scale (NPPB) was developed by Hu J in 2013 [6]. A total of 29 items were included in the questionnaire, which was divided into 5 dimensions:, including personal growth (6 items), good nurse-patient relationship (6 items), recognition from families and friends (7 items), positive professional perception (5 items), and a sense of belonging to a work team (5 items). On a 5-point scale, the responses ranged from 1 (completely do not agree) to 5 (completely agree). The total score was between 29 and 145 points. A higher score indicates a better NPPB. The Cronbach’s alpha coefficient was 0.958. The subscale coefficients ranged from 0.821 to 0.893.

### Data analysis

The scores for general self-efficacy, POS, NPPB and NPE and the descriptive statistics and the Pearson correlations for all study variables were analyzed using SPSS (version 23.0, IBM).

**Fig. 1:**
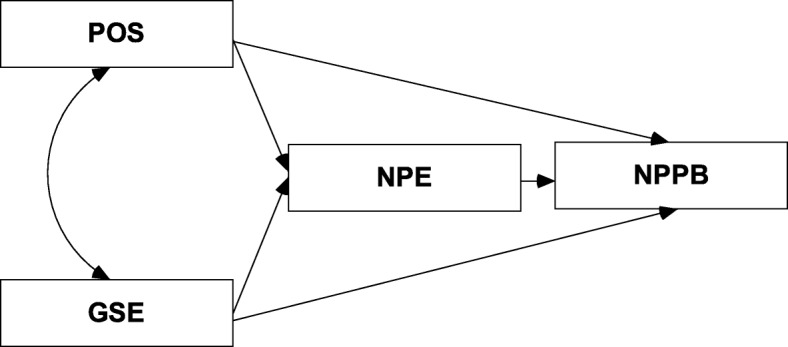
Hypothesized theoretical model of POS, GSE, POS and NPPB. Note. POS=Perceived Organizational Support; GSE = General Self-efficacy; NPE = Nursing Practice Environment; NPPB = Nurses’ Perceived Professional Benefits

The proposed path model of general self-efficacy, POS, NPPB and NPE outlined in Fig. 1 was estimated using the analysis of moment structures (AMOS, version 23.0). Path model analysis with maximum likelihood was used to verify the relationships and predictions with the assumption that the multivariate data of general self-efficacy, POS, NPPB and NPE were normally distributed. Measurement errors of the path model analysis were also considered.

The goodness of fit standard for path model analysis was judged by the absolute fitness index (*X*^*2*^<3.84, RMR<0.05, RMSEA<0.05, GFI>0.09, and AGFI>0.09), value-added fitness index (NFI>0.90, RFI>0.90, and CFI>0.90), and simple fitness index (PGFI>0.50, PNFI>0.50, PCFI>0.50, and *X*^*2*^*/df* <2.00). It is necessary to modify the path model to improve the model fit when the modification indices are larger than 4. Furthermore, mediating effects were tested by investigating standardized indirect effects, and the bootstrapped confidence interval estimates were calculated to confirm the significance of the indirect effects [39, 40].

## Results

### Description of respondents

The demographic and clinical characteristics of paediatric nurses are presented in Table 1. The sample consisted of 300 nurses. Of the respondents, 99.0% were female (*n* =297), 58.30% were under 30 years old, 78.70% were contract nurses (contract nurses refers to the employer and the employee determining the relationship by the contract), and only 21.30% of the participants were official nurses (official nurses refers to national nurses, and their basic salary and local subsidy are allocated by national financing). In China, official nurses are in a better position than contract nurses in terms of job stability, salary payment, salary adjustment, retirement and insurance benefits. Of the respondents, 55.0% have served as nurses for 1 to 5 years, 97.6% were below a college degree in terms of their current academic status, 53.30% were married, 62.0% were not in infertility situations, and 39.30% had an income status of 12,000-15,000 yuan (per month).

**Table 1:** Demographic and clinical characteristics of the sample (*N* = 300)

Variable	n(%)
Gender	
Male	3(1.00)
Female	297(99.00)
Age, years	
≦30	175(58.30)
≧31	125(41.70)
Appointed form	
Official nurses	64(21.30)
Contract Nurses	236(78.70)
Years of service.	
1–5	165(55.00)
≧5^+^	135(45.00)
Current academic status	
≦College degree	293(97.6)
≧Bachelor degree	7(2.30)
The professional status of nurses	
Junior	265(88.3)
Mid-level	33(11.0)
Senior	2(0.7)
Marital status	
Married	160(53.30)
Unmarried	140(46.70)
Fertility situations	
No	186(62.00)
Yes	114(38.00)
Income status (per month)	
≦12,000 yuan	104(34.60)
12,000–15,000 yuan	118(39.30)
> 15,000 yuan	78(26.00)

*Abbreviation*: *n* Number, *SD* Standard deviation

Means, standard deviations, and correlations among the related variables are presented in Table 2. The mean scores of the POS scale denoted a moderate level (M =48.73, SD = 5.74), but the mean score of general self-efficacy was low (M =24.88, SD = 4.39) when considering the judgement criteria of Zhu (2016) [41]. The mean NPE score was 91.33 (SD = 9.04). The average level for NPPB was 114.24 (SD = 7.48).

**Table 2:** Correlations among POS, GSE, NPE and NPPB (*N* = 300)

Variable	Mean	SD	POS	GSE	NPE	NPPB
POS	48.73	5.74	1			
GSE	24.88	4.39	.576^**^	1		
NPE	91.33	9.04	.550^**^	.457^*^	1	
NPPB	114.24	7.48	.662^**^	.455^**^	.580^*^	1

*Abbreviations*: *POS* Perceived organizational support, *GSE* General self-efficacy, *NPE* Nursing practice environment, *NPPB* Nurse’s perceived professional benefits, *SD* Standard deviation

**P < 0.01, *P < 0.05

### Correlations among study variables

The results of the correlation analysis, such as general self-efficacy, POS, NPE and NPPB, were significantly correlated with each other; NPE and POS, POS and NPPB, and NPE and NPPB all had the best relationships. Therefore, a multivariate path model was conducted to test the relationship mediated by NPE.

### Path analysis

**Fig. 2:**
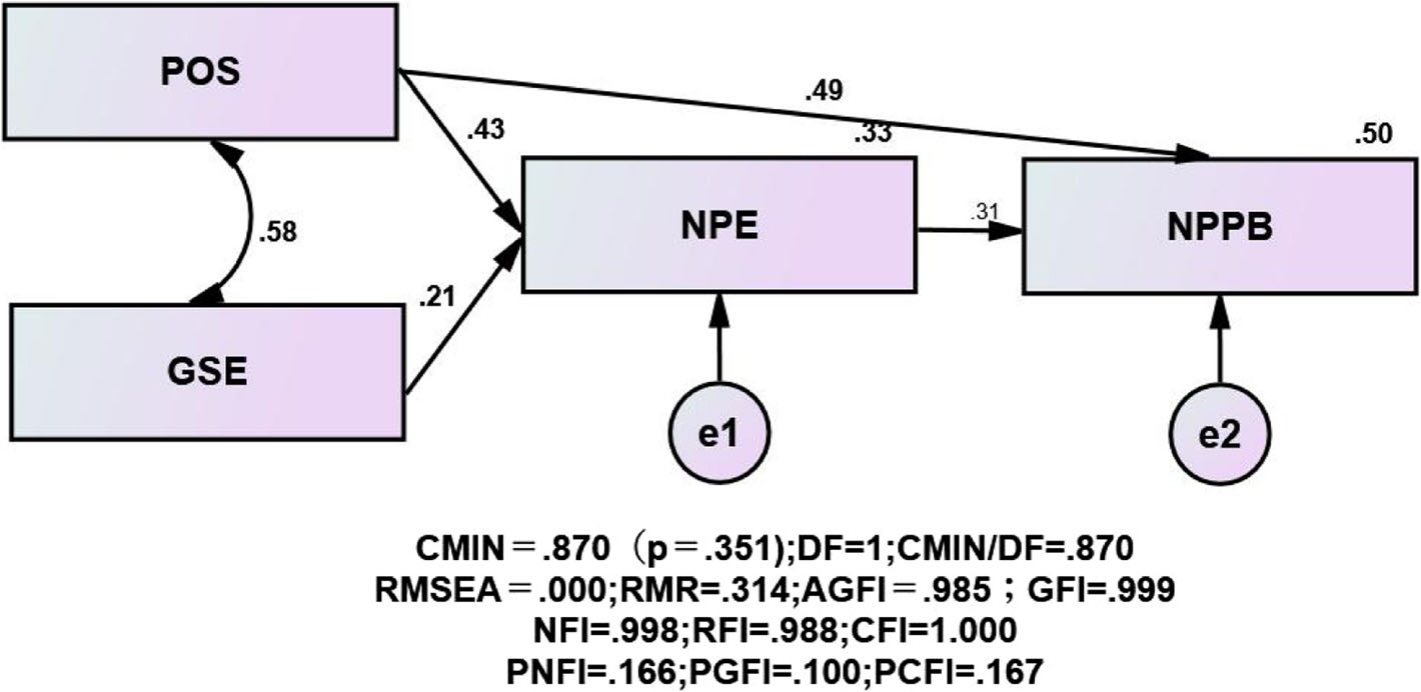
The final mediating effect model of NPE Among GSE, POS and NPPB (*N*=300). Note. Abbreviations: POS=Perceived Organizational Support; GSE=General Self-efficacy; NPE=Nursing Practice Environment; NPPB= Nurses’ Perceived Professional Benefits. Four variables are connected by significant paths.The numbers on the straight arrows indicate the standard path coefficients. POS and GSE are connected by bidirectional arrow curves, and the number on the line indicates the correlation coefficients

The results of the proposed model showed that there was no negative variance and a large standard error in model, so the model did not violate the identification rules. This model’ s fitness and the actual data were good from the whole index of the mode. The absolute fitness index, value-added fitness index and simple fitness index all met the adaptation standards (Fig. 2).

Although the RMR did not meet the standard of less than 0.05 in this study, the smaller the RMR was, the better the model. Because the RMR is easily affected by the measurement variables’ unit, it often shows inconsistent data [30].

The study found that all the paths with POS, GSE, NPE and NPPB were significant in the model by testing the effects of the explanatory variables on the response variables in Table 3. POS had significant direct effects on NPE (*β*=0.677, *P*=0.000) and NPPB (*β*=0.640, *P*=0.000), NPE had significant direct effects on NPPB (*β*=0.257, *P*=0.000), and GSE had significant direct effects on NPE (*β*=0.430, *P* = 0.000). POS (*β* = 0.174, *P* = 0.000) and GSE (*β* = 0.111, *P* = 0.000) significantly influenced NPPB indirectly through NPE mediating the relationship.

**Table 3:** Indirect and direct effects of explanatory variables on response variables (*N* = 300)

Structural Path	Non-standardized Coefficients	Standard Coefficients	*P*	95% CI
Direct effects				
POS → NPE	0.677	0.430	0.000	0.497 to 0.857
POS → NPPB	0.640	0.491	0.000	0.514 to 0.765
NPE → NPPB	0.257	0.310	0.000	0.177 to 0.366
GSE → NPE	0.430	0.209	0.000	0.195 to 0.665
Indirect effects				
POS → NPPB	0.174	0.133	0.000	
GSE → NPPB	0.111	0.065	0.000	

*POS* Perceived organizational support, *GSE* General self-efficacy, *NPE* Nursing practice environment, *NPPB* Nurse’s perceived professional benefits. Arrow direction(→) represents the effects of the explanatory variables on the response variables. Indirect effects:0.174(POS→NPPB) = 0.677 × 0.257; Indirect effects 0.111(GSE→NPPB) = 0.430 × 0.257

## Discussion

The direct effects of POS and NPE on NPPB were further confirmed; meanwhile, the direct effects of POS and GSE on NPE were also found, which provides initial evidence for the mediating the role of NPE in the effects of POS and GSE on NPPB. These findings indicate that a better way to improve NPPB in paediatric nurses needs to be addressed.

Most studies have reported that improving POS can reinforce NPPB [27, 29]. Nurses can effectively exert subjective initiative and improve their confidence, self-worth and job satisfaction and thus produce a positive psychological effect [42]. Moreover, when POS meets the nurses’ emotional needs, a sense of responsibility and emotional commitment to the organization is likely to be produced, and their turnover tendency can therefore be reduced [20, 43, 44]. This also proves the results of POS on NPPB from another aspect.

However, there is limited research on how POS improves NPPB; in this study, NPE and all of the dimensions are positively correlated with NPPB. By allowing nurses to participate in hospital affairs, providing sufficient human and material resources, ensuring high-quality nursing services, improving nurse leader’s ability and leadership style and enforcing doctor-nurse cooperation, nurses can increase NPPB. Therefore, we believe that POS must be stimulated in a certain environment to produce certain benefits. An international study has found that hospitals with good work environments and nursing staff improves nursing outcomes, and the quality of the good hospital work environment included perfect managerial support for nursing care, good doctor-nurse relations, nurses engaging in decision making and organizational priorities on care quality [45]. Other researchers have all reported that NPE had a significant bearing on job satisfaction, intention to leave a current position, job retention, job burnout, job-related stress and anxiety [46, 47]. The good NPE listed above could provide nurses with the opportunity to expand job skills and informal networks and increase their knowledge of the system. This leads a better awareness of perceived organizational support and encourages nurses’ motivation to succeed and enhance their commitment [48, 49]. In summary, a good practice environment makes nurses accept and cherish their job and positive emotions develop, such as attachment to the organization, gratitude, a sense of security, and a sense of responsibility. As a result, nurses get paid more for their work and give more in return for their pay, which forms a virtuous circle.

In this study, general self-efficacy had no direct effect on NPPB. Several studies have reported a correlation between general self-efficacy and NPPB [50, 51], but these previous studies only tested two variables and were aimed at nursing interns or nursing students who were at the early stage of their internship. In these studies, other factors were not taken into account, particularly the nursing practice environment, working hours and especially the paediatric practice environment. Therefore, in this study, we found no direct effect between general self-efficacy and NPPB. However, general self-efficacy had an effect on the nursing practice environment, which has been demonstrated in many studies [52]. On the one hand, nurses with a high level of self-efficacy can better perceive the benefits of the nursing practice environment and can make full use of the benefits of the environment for themselves. Therefore, their POS is also at a high level, and the correlation between POS and general self-efficacy was confirmed in this study. On the other hand, they can also avoid the disadvantages of the environment and find appropriate solutions, thus not only promoting self-adaptation and development but also promoting the development of nursing work. Above all, a good nursing practice environment could be regarded as a moderating variable, increasing staff's perceived professional benefits. For successful implementation, nursing administrators should implement effective strategies to promote a comfortable practice environment [6, 53].

## Implications for practice and policy

Based on the findings of this study, it is possible to indicate some implications for future clinical practice and policy. First, perceived occupational support had a stronger direct effect on nurses’ perceived professional benefits, which suggests the value and role of perceived occupational support. As a nursing manager, we should take measures to establish and improve welfare mechanisms, such as to improve the work system, leave system, scheduling system, and welfare system of paediatric nurses, to reduce compassion fatigue by reinforce personality traits, to maintaining the habit of engaging with outdoor activities [54]. Hospital administrators may strengthen family support and social support for pediatric nurses. For instance, a psychological counseling department should be set up to regularly realize paediatric nurses’ needs. Second, the nursing practice environment played an intermediary role between perceived occupational support and nurses’ perceived professional benefits, so strategies should be implemented to optimize the nursing practice environment, focusing on improving paediatric nurses’ professional skills, communication skills and reducing the rate of violence and unhealthy phenomena at work. Finally, the findings showed that self-efficacy was prominently associated with the nursing practice environment, perceived occupational support and perceived professional benefits.

Thus, paediatric nurses should develop efficient programmes to improve self-efficacy. Meanwhile, an emphasis should be placed on the nursing professional development for paediatric nurses, including the clinical decision-making patterns for pediatric nurses. And hospitals should provide nurses with more opportunities for further study and to improve their confidence and satisfaction in their work.

## Limitations

This study has both strengths and limitations. A path analysis was used to explore the moderating effects of NPE among GSE, POS and NPPB for paediatric nurses, implying that some leaders could increase nurses’ perceived professional benefits by improving specific environmental measures.

Regarding limitations, first, convenience sampling (from Class A tertiary hospitals) might limit the generalizability and robustness of the conclusions of this study. Therefore, nurses from primary and secondary hospitals should be included in our research in the future to verify our hypothesis and model.

Second, the questionnaires might not truly reflect the thoughts of paediatric nurses due to the self-reported nature of the study. Future studies should consider both subjective and objective data on GSE, POS, NPPB and NPE. Finally, our conclusions are based on a cross-sectional database, which are unable to highlight the direction of causality of some factors, therefore, a longitudinal research large-scale investigation would be necessary. Nevertheless, we feel that the limitations do not nullify our conclusions.

## Conclusions

This study tested the relationships among general self-efficacy, POS, NPE and NPPB and confirmed the moderating effect of NPE on the relationships among general self-efficacy, POS and NPPB in paediatric nurses. This finding implies that some hospital managers should pay attention to the importance of general self-efficacy and POS to reduce job burnout and increase nurses’ retention.

Meanwhile, effective measures should be taken to improve nurses’ practice environment in hospitals to raise nurses’ enthusiasm and confidence in their work. However, the study may not be applicable to junior nurses or trainee nurses, but our model can be used to improve a NPPB model for nurses increasing job satisfaction and as a foundation for the improvement of appropriate nurses’ practice environment and interventions.

## Data Availability

The datasets used and/or analyzed during the current study are available from the corresponding author on reasonable request.

## Abbreviations

AGFI: Adjusted goodness-of-fit index
CFI: Comparative fit index
DF: Degrees of freedom
GFI: The goodness-of-fit index
GSE: General self-efficacy
NFI: Norm fit index
NPE: Nursing practice environment
NPPB: Nurse’s perceived professional benefits
PCFI: Parsimony comparative fit index
PGFI: Parsimony goodness-of-fit index
PNFI: Parsimony-adjusted norm fit index
POS: Perceived organizational support
RFI: Relative fit index
RMR: Root mean square residual
RMSEA: Root mean square error of approximation
SD: Standard deviation
SEM: Structural equation model
*X*^*2*^*/DF*: Likelihood ratio
